# The Interactional Structure of Accounts During Small Group Discussions Among Autistic Children Receiving Special Education Support in Finland

**DOI:** 10.1007/s10803-023-05916-9

**Published:** 2023-02-07

**Authors:** Juliene Madureira Ferreira, Kristen Bottema-Beutel

**Affiliations:** 1 Faculty of Education and Culture, Tampere University, Tampere, Finland; 2 Lynch School of Education and Human Development, Boston College, Boston, USA

**Keywords:** Autism, Social interactions, Agency, Social competencies, Participation

## Abstract

Through a conversation analytic approach, we investigate the emergence of accounts provided by autistic children in small-group discussions. Nine Finnish children (7–10 years old) attending school with special support participated in a five-month-duration pedagogical practice purposefully designed to enhance children’s participation in groups. We analyzed videos of sharing circles where children discussed their ideas and interests. Our data show three different account structures, which created different modes of children’s participation and gradually changed how they positioned themselves in the group. Results show how accounts can create different focuses of attention; reveal children’s reflections on what is relevant to them, and how to promote the exchange of ideas within a small group. Implications for the development of educational practices are discussed.

**Supplementary Information:**

The online version of this article contains supplementary material available 10.1007/s10803-023-05916-9.

## Introduction

Inclusive educational practices have come a long way since the Salamanca Declaration in 1996 and, in many aspects, today’s world may look more accepting and open to the participation of students with disabilities than thirty years ago (Ainscow, 2016; European Agency for Special Needs & Inclusive Education, 2018). However, social interactions between autistic and non-autistic students in classrooms can be challenging for both groups of students and can mean that autistic children have fewer opportunities for classroom interaction with peers (Fasano et al., 2021).

Although much autism research has focused on enumerating what is perceived to be communicative deficits of autistic people, several studies have approached this phenomenon aiming at understanding autistic sociality; the unique repertoires that autistic people bring to social interactions (Ochs & Solomon, 2010; Sterponi, 2017; Sterponi & Chen, 2019; Sterponi & Shankey, 2014). Instead of comparing communicative performances against ‘standard’ non-autistic interactions, the idea is to characterize the unique linguistic resources autistic people bring to interactions and see how they are deployed in service of interactional aims (Bottema-Beutel, 2017; Sterponi et al., 2015). Understanding autistic sociality may be the most effective and respectable way to guarantee autistic people’s participation in social contexts (De Jaegher, 2021). Aligned with this idea, the present study explored how autistic children elaborated on and used ‘accounts’ in small-group discussions. We aimed to understand how accounts of their and others’ behaviors, ideas, and interests emerge and are articulated to create children’s participation in educational contexts.

### Accounts in Dialogues and Their Relevance to Social Interactions

Accounts are broadly understood as explanations people provide to justify actions, requests, or arguments in daily social interactions with others (Antaki & Fielding, 1981). They are commonly observed in a dialogue to “excuse, justify or otherwise exonerate the speaker from socially sanctionable behavior” that go beyond causality (Antaki, 1994, p. 43), appearing both in unexpected, controversial, or problematic actions of the speaker (Heritage, 1988) or to display an explanation of why a preferred action can or cannot be performed (Levinson, 1983). For example, accounts can appear spontaneously when one justifies their behavior, anticipating it might be controversial in the interaction context, e.g., “The garbage needs to be taken out, but I will do it in the morning; It is too cold now.” Alternatively, accounts can be solicited by others, as in the following scenario:

Speaker A: Can you grab coffee?

Speaker B: Not today.

Speaker A: Why not?

Speaker B: I already have plans.

Accounts allow us to sustain engagement and manage social relationships (Heritage, 1988) by mitigating responses to unexpected or dis-preferred conduct. Knowing when to account for behaviors, ideas, or actions, as well as identifying an account provided by others is an important conversational skill that supports maintaining reciprocity in the interaction.

In educational contexts, accounts are often encountered within student–teacher interactions following requests (Antaki, 1994), and they are important structures in social dynamics that support both learning and social interactions. For example, participating in the explanatory discourse during shared reading enables pupils to practice providing explanations for events or behavior that transcend their own experiences (Gosen et al., 2013). Therefore, investigating how children account for their ideas and behaviors provides insights into how they are making sense of the world around them, and how they are building their sense of agency in this same world.

In non-autistic children, this skill is clearly developed in early childhood and becomes more sophisticated throughout development. Children master the formal structural aspects of conversation, such as turn-taking, much earlier than the pragmatic-functional aspects, such as coherence and relevance (Blum-Kulka et al., 2010). However, for autistic children, conversational skills involving complex pragmatic-functional structures, such as accounts are potentially challenging (American Psychological Association [APA], 2013) as they could be conceptualized as relying on an ability to understand others’ mental states. According to leading theories on human cognition (e.g., Theory of Mind and Simulation Theory, see Sally & Hill, 2006), social interactions are explained by humans’ capacity to ascribe mental states (e.g., beliefs, thoughts, feelings, intentions, and desires) through inferential processes. These inferences are built either because of the similarity of human minds; by analogy and first experience, we can represent others’ minds (Theory of Mind), or by the ability to mentally simulate others’ feelings, emotions, and intentions (by putting oneself in others’ shoes) (Simulation Theory). In any case, under these theoretical frameworks, autistic people are proposed to lack intersubjective skills and perform poorly in social interactions requiring these sophisticated processes. Thus, conversations with autistic people have been characterized as involving short responses, sporadic initiations, and infrequent sharing of new, relevant information. These conversational characteristics are believed to hinder opportunities to learn from and participate in social interactions, increasing the risk of social isolation (Koegel et al., 2003), demanding auxiliary instructional intervention to improve social conversation (Hughes et al., 2012), and preventing children’s participation and achievement in the regular school context. For this reason, autistic children are often advised to learn from smaller group settings, supported by one-by-one teaching and flexible planning that can be adjusted to their developmental differences. Theories explaining social interactions through the idea of hidden intention and sophisticated intersubjective dynamics, as well as methods based on assessing the complexity of the speakers’ syntax (Price-Williams & Sabsay, 1979), still guide most research and practices that address issues related to autistic communication.

However, other theoretical views center on social interaction instead of relying solely on social cognitive explanations for behavior. For instance, an enactive view proposes that cognition is embodied and non-trivially dependent on the body and enacted (brought forth) through actual engagement with the environment (De Jaegher, 2021; Di Paolo et al., 2018). According to this framework, what drives and maintains the interactions is the principle of self-organization and the fundamental need to enact with the world (including others), not our ability to simulate or predict others’ minds. The crucial aspect of this theoretical framework is not to say that we don't mentalize; surely, we do, but it is to say that in the process of interacting with others, our bodies (and their enactions) have a predominant role in shaping reality. Indeed, investigations of autistic children's accounts using similar frameworks have shown that these children do indeed show evidence of a practical orientation to their interaction partner’s interpretations of their behavior (Henderson, 2019).

Under this perspective, the most important methodological innovation must be to look at the interactions as dynamic living organisms, allowing them to reveal their own life. This means, in the case of autistic interactions, to look at it from the perspective of what participants bring to the dialogue instead of what non-autistic people would understand as relevant to the interaction. It is a new paradigm, challenging our understanding of the communication competencies of autistic people (Fantasia et al., 2014). The present study corroborates this approach; mainly, by creating a setting for autistic interactions to be analyzed.

### Participation and Small-Group Discussions Among Autistic Children

In inclusive education, the principles of participation and achievement are guiding elements in elaborating pedagogical practices. The participation framework in the context of inclusive education is a complex concept to define, and it is pointed out as one of the main shortcomings of inclusive policies and practices (Black-Hawkins et al., 2007; European Agency for Development in Special Needs Education [EADSNE], 2011). Beyond the idea of physical presence in the classroom, participation entails the interactions established with others, the engagement in the learning process, and the meaning that schooling will have for each child (Ferreira, 2017). In a general manner, participation is understood as the quality of children's experiences whilst they are in school and, therefore, must incorporate the views of the learners themselves (Ainscow, 2016, p. 147). Consequently, the concept of achievement will entail looking at what the child is capable of experiencing in school, going beyond identifying standardized, pre-defined learning outcomes across a universal curriculum. Achievement is defined by the developmental goals in a holistic perspective, and it is not restricted to the results extracted from test or examination marks (Aiscow & Messiou, 2021; Ferreira 2017). Therefore, in the analysis of participation and achievement of autistic students, one must consider the singularity of the developmental processes and their unique learning needs. This often demands an individualized learning plan, tailored social interactions, and more hours of one-to-one teaching, enhanced in small-group teaching and learning sessions.

Small-group discussions constitute different learning settings compared to teacher-fronted activities or one-to-one lessons where the teacher’s role is prominent. Small-group discussions are effective for developing complex thinking (Applebee et al., 2003; Murphy et al., 2009), enhancing text comprehension (Maine & Hofmann, 2016), offering opportunities for students to develop collaborative (Chinn, et al., 2001) and individual reasoning skills (Mercer, 2000). All because small-group interactions facilitate the students to talk and think together, scaffolding multiple directions for the conversation (Van der Veen et al., 2015) and balancing out the benefits of a structured learning setting with a more flexible and varied composition of discursive elements (i.e., that are not reduced to teacher-controlled IRE-sequences) (Soter et al., 2008). The students are free to select in turn another student as the next speaker, creating a situation where the epistemic domain is divided among different participants. As a result, the dominant turn-taking pattern would ideally no longer have the Teacher-Student–Teacher-Student order omnipresent in teacher-fronted classroom interaction, but rather an order that reflects the multiparty character of these discussions: T–S–S–S (Myhill 2006). Our study focused on unraveling the dynamics of small group participation frameworks in the context of special education. It is important to consider that although the indication of special support is, in many cases, a common intervention for the better learning and development of the child, the ideal situation from an inclusive perspective is that all learning, including supporting the development of social and communicative skills happens in the regular classroom (Ainscow & Messiou, 2021).

### The Present Study and Its Analytical Framework

The present study is part of larger project “Embodiment, (inter)subjectivity and the construction of children’s agency in learning (2021–2023)”^1^ conducted by the first author, through which the construction of the sense of agency in learning is examined amongst children that are receiving special education support in Finland. What we present at this time is the qualitative analysis of small-group discussions in which autistic children participated. The aim was to understand how children made meaning of their experiences and participated in small-group discussions. We analyzed particularly the sequence organization (i.e., how turns-at-talk are ordered and combined to produce social action involving accounts provided by children during sharing circles. Different from other actions in conversation, such as requests or complaints, accounts demand awareness of others' expectations and a time-sensitive response to them within the conversation. The following research questions guided the investigation:

1. What types of accounts are present in the sequence organization of autistic children when talking about their work and ideas?
2. How do accounts emerge and develop to support participation in small-group discussions?

To answer these research questions, we build upon previous literature by applying conversation analysis (CA) as our method of research (Sidnell & Stivers, 2013). CA focuses on the “analysis of sequences of interactions and of turns with sequences” (Heritage, 1988, p. 129) as well as participants' interactional moves in their turns at talk (Hutchby & Wooffitt, 1998). This method explores how each statement is connected to previous ones and how participants understand and respond to one another, unraveling how sequences of actions and meaning-making are generated (O’Reilly et al., 2016).

## Methodology

### Participants and Site of Research

Participants in this study were nine Finnish children (one with a multicultural background, the father is American) aged from seven to ten years, regularly attending school under the regime of part-time special support, in the Pirkanmaa region, Finland. Students belonged to a special classroom composed by twelve children in total. All students involved in this study were either diagnosed with ASD or under investigation to conclude the diagnosis according to criteria of the ICD-10 (World Health Organization [WHO], 1992) or DSM-V (APA,^ 2013^). Although all children participating in the study did not present additional intellectual disabilities and could usually communicate verbally, they often could not engage in school activities involving written or spoken discussions, thus demanding special support. Special support is the third and final level of the three-tier educational support service offered to students in the Finnish Inclusive Education System (see, EDUFI, 2016; Ferreira et al., 2022). Special support can be delivered through different actions such as material adjustments, part-time small-group or individualized lessons with special education teachers, or a flexible curriculum (OSF, 2020). For the participants in this study, special support was carried out in a special classroom within a regular school. Most of the lessons are organized in small groups (from 5 to 12 children per class), and for specific subjects such as second language learning, children are placed in larger groups with children that do not demand special support.

Special classrooms within mainstream schools are uncommon in Finland. Thus, the recruitment process was purposeful; we solicited information from the municipality to find classrooms that fit the study’s requirements (small groups with autistic children) and contacted schools’ coordinators directly. Two schools were contacted, and one accepted to participate in the study. Subsequently, we invited the teacher and children to participate.

### The Idea Diary as a Pedagogic Method to Support Small-Groups Interactions in Special Education Support

The Idea Diary as a pedagogic method was elaborated by Muniz (2015, 2020) to provide a forum through which students could bring their own ideas, interests, and knowledge about their experiences in different social contexts to the school context. It is grounded in the action tripod ‘experiencing—writing—sharing’, where children are invited to reflect on and keep a diary of their learning experiences, current interests, and important events in their lives and share these memories with the class. This activity framework supports teachers in systematically accessing and understanding the subjective productions (i.e., how children make meaning) emerging from students’ learning process, supporting creative learning (Muniz & Mitjáns-Martinez, 2019). Teachers and children establish a dialogical relationship and equally participate in producing knowledge about children’s experiences and interests.

**Fig. 1:**
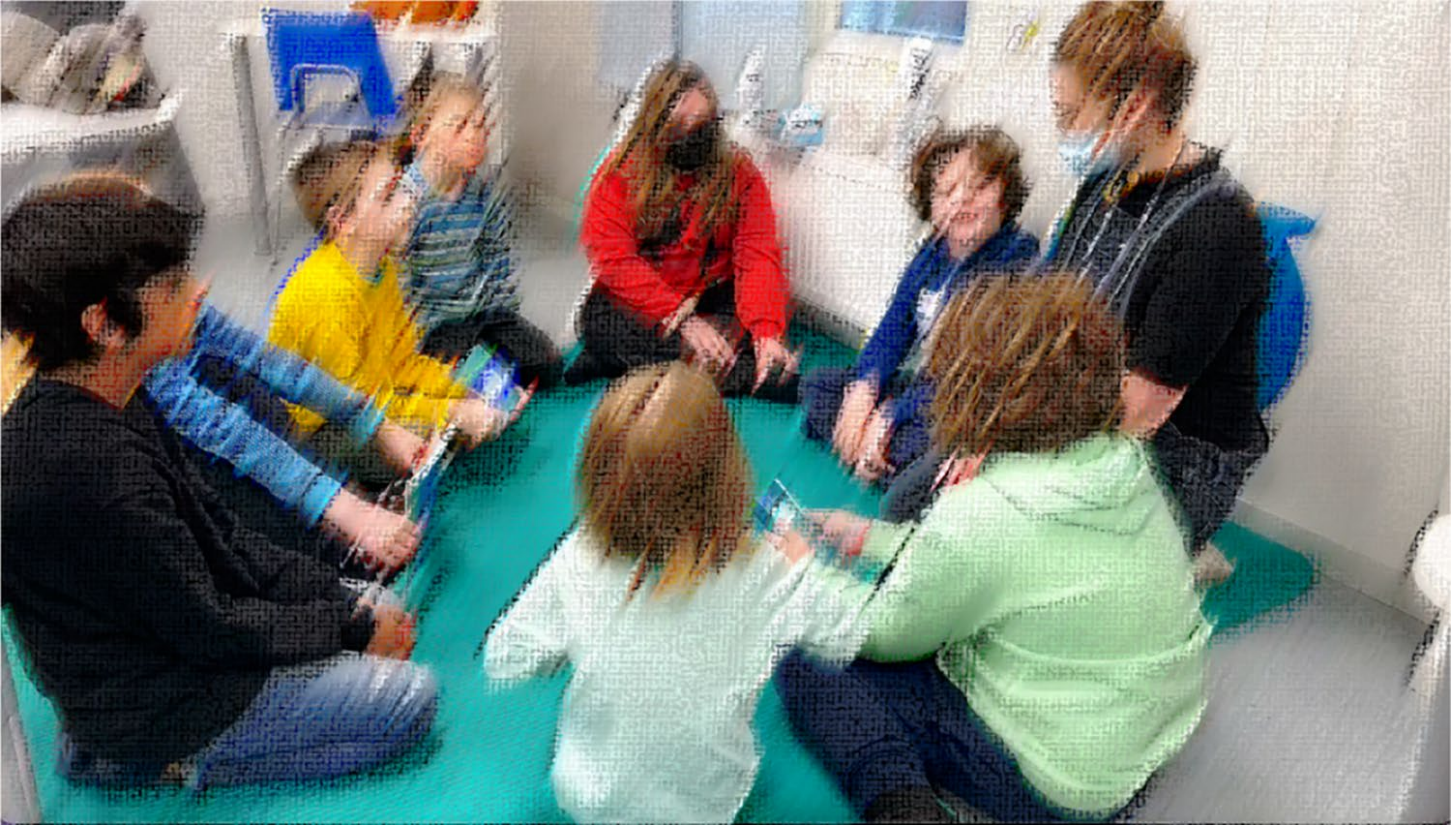
Illustration of a sharing circle. Picture retrieved from the first author’s archive. A filter is applied for identity protection

Two fundamental activities structure the Idea Diary, first is the sharing circle. The teacher invites the children to share what they have produced in their diaries in moments called sharing circles (see Fig. 1). The sharing circles consist of small group discussions about the content in the diary. Sharing circles are organized and conducted by the teacher; during this moment, children present their productions (e.g., writings, drawings) and talk about their experiences. The teacher’s role is to support children in expressing themselves.

The sharing circles offer a unique conversational structure that combines a routinized interaction, i.e., all sharing circles are organized and conducted similarly, with the spontaneity of child-centered discussion. This structure is designed to support the development of children’s subjectivity (Muniz, 2020). Unlike classroom interactions where the pedagogical questions are known to the teachers who pose them (e.g., known-answer or test questions; see Grosse & Tomasello, 2012; Schegloff, 2007), in the dynamic proposed in the Idea Diary the epistemic authority belongs to the child (Heritage, 2012). Even though the Idea Diary was not initially designed for a particular target group, previous experience with the tool showed great potential to benefit the learning processes of autistic children (Lima et al., 2022). Sharing circles can organize the interaction, facilitating children’s participation and promoting gradual development in interacting with others. Therefore, we understand that the Idea Diary as a pedagogic method scaffolds social dynamics that build mutual trust and progressivity (Sterponi & Fasulo, 2010).

The second activity grounding the Idea Diary is incorporating children’s experiences into the classroom curriculum. The teacher then uses this knowledge, weaving children’s interests into the school curriculum and potentializing creative learning in the classroom (detailed methodology of the Idea Diary is available on the website http://www.lucianamuniz.com.br/metodologia/). The Idea Diary creates materials and space in the classroom routine for investigating children’s reading/writing of the world, interweaving formal learning objectives with children’s life experiences. The Idea Diary provides opportunities for children to understand reading and writing as processes of communication, production, and self-expression (Muniz, 2020). Participation, in this perspective, is identified by how children construct the Diary (producing material) and gradually incorporate references from the collective work being constructed. Achievement is observed and assessed considering transformations in participation by comparing intrapersonal changes along a timeline of events (Lima et al., 2022).

To implement the Idea Diary, teachers are trained in the method. The training entails two four-hour modules where the teacher will be introduced to the theoretical framework and presented with step-by-step video materials explaining the method, including guidance on mediating the small-group discussion to promote children’s participation. Examples of Idea Diaries are also provided, and a case study is discussed. The training aims at building a new theoretical and methodological repertoire for a student-centered approach, which the teacher can incorporate into their pedagogical approach as they judge fit.

### The Data and Its Analysis

Our dataset was derived from the project database referred to previously and consisted of 11 video-recorded sharing circles of the Idea Diary sessions, where students and their teacher discussed children’s diary entries. The duration of sharing circles averaged 25 min (a total of 240 min of video data). The sharing circles were recorded once a week throughout the Spring 2021 academic semester, according to the teacher’s schedule. Videos included only the discussions over the idea diaries. To ensure the quality of data gathering, the first author was present during the data collection and used two cameras, resulting in synchronized videos in which the teacher and all students were visible simultaneously.

In the first step of the analysis, all 11 recordings were transcribed *verbatim* in Finnish. Next, transcripts were translated into English by the first author and double-checked and back translated by two native speakers, and an English gloss was included under each line of Finnish and marked in red. The first author and a research assistant scanned the text for sequences of adjacency pairs containing accounts. Adjacency pairs are a two-part exchange in which the second utterance is functionally dependent on the first. Sequences containing accounts are identified by an explanation usually provided in the second turn; it should justify rejecting or refuting the invitation or request in the first turn. For example, in the dialogue below, John justifies his inability to join Mary for lunch after rejecting the invitation (marked by the arrow).

Speaker A: I am making pie for lunch today; would you care to join us?

Speaker B: I don’t think I can make it today.

  I…I am running late this morning.

This analytical process focused on identifying all sequences containing accounts expressed by children across their dialogues in all sharing circles. Subsequently, the second author double-checked the structure of all selected sequences ensuring 100% agreement, which was reached by consensus. The second step of analysis consisted of interpreting the context of the accounts. The first and second authors created a codebook reflecting four types of accounts, which reflect all possible combinations of the initiator of the account (self or other) and the provider of the account (self or other): self-initiated, self-provided; self-initiated, other-provided; other-initiated, self-provided; and other-initiated, other-provided. The codebook consisted of general examples of each of the types of accounts. It was used as a reference for the analysis of individual sequences. Finally, a modified version of CA conventions (Jefferson, 1986, see appendix A for the conventions) was applied to the Finnish portion of the selected sequences and then transferred with adjustments for the English gloss. Each transcript underwent two rounds of ‘checks’ by the research team to verify transcription accuracy. It was important to apply the conventions primarily on the Finnish transcript to ensure that the analysis was properly culturally and linguistically contextualized. Additional components of each interactional turn that appeared relevant to the participant’s ability to make meaning from the interaction were also examined, including gaze, gestures, and bodily actions (figures are supplied for many extracts below to draw attention to these features as appropriate). The final analytical agreement was done through constant discussions between the two authors and their teams. The names of the students and teachers were anonymized; each child was denominated by a number, e.g., child 1, child 2, etcetera.

The accounts appeared in different sequence organizations, all, however, in a turn regularly preceded by an orientation to an object of joint attention (e.g., the idea diary of the child presenting their work to the class). By analyzing children’s engagements in parallel to the types of accounts provided it was possible to understand the different modes of participation created during the sharing circles. The analysis uncovered patterns of interaction that recurred across the collection as a whole. Therefore, the fragments shown in this paper can be considered representative of the collection of accounts. These detailed transcripts also enable readers to verify the claims made by the analysts.

### Ethical Considerations

Ethics approval for this study was gained from the Tampere Municipal Institutional Review Board before the commencement of the study (protocol under the name of the first author). Written informed consent for the study was obtained from the participating school, teachers, guardians, or parents of all participating children. Special attention was given to assuring children were aware of and in agreement with the study. Before data collection, the first author took part in weekly classes, meeting the students, establishing rapport, and introducing the video equipment to the children. During this time, the researcher explained the proceedings and, with the teacher’s support, inquired about the children’s thoughts concerning participating in the study.

Consent was denied for two out of eleven children. To respect parents’ and child’s decisions, we scheduled video recordings when the two students were absent from the class. Children not engaged in the study were, however, participating in the other activities of the Idea Diary, and were included in all learning opportunities that derived from the shared discussions.

## Results

**Fig. 2:**
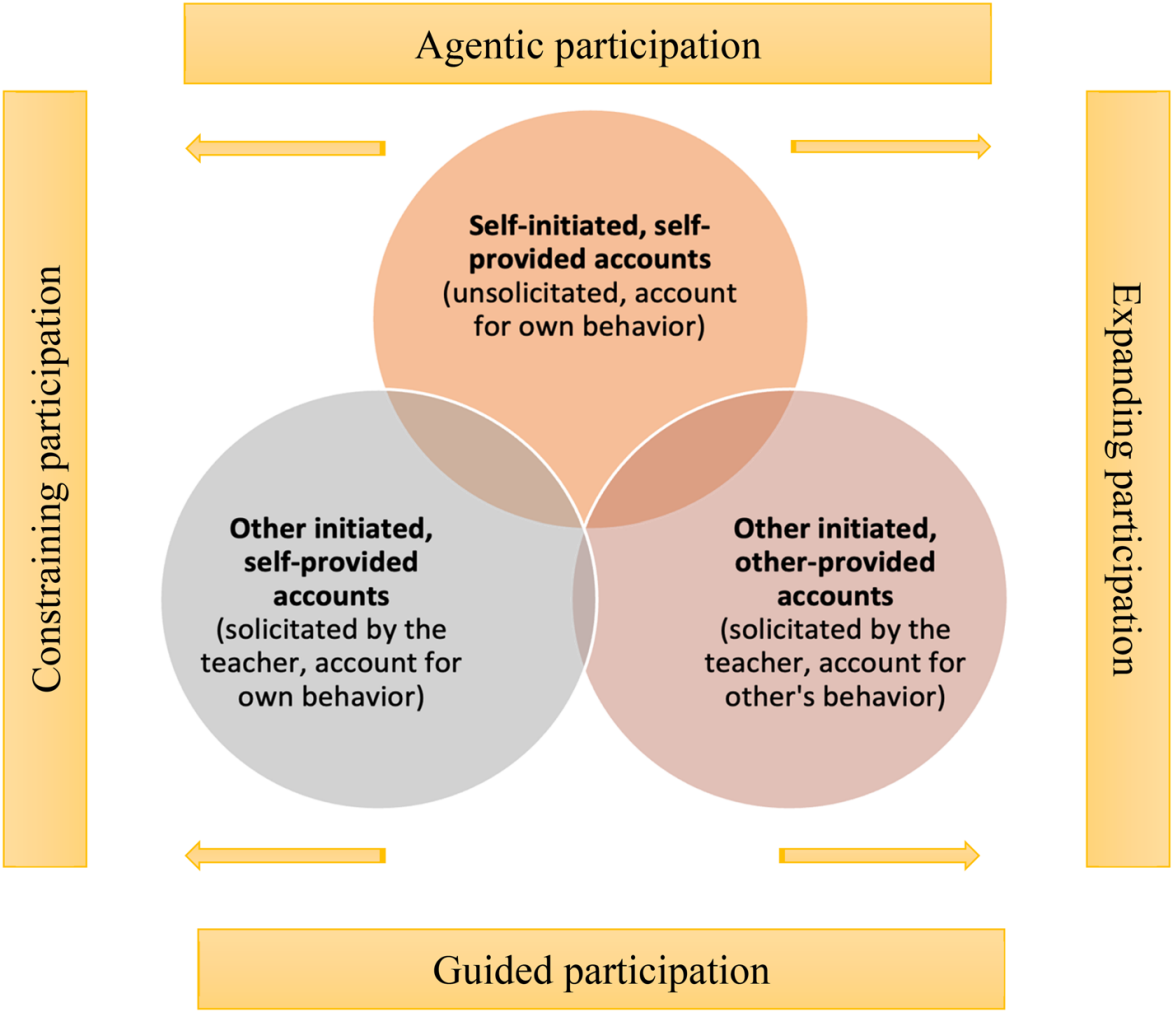
Types of accounts and modes of participation

Our collection of accounts consisted of 124 episodes. Accounts appeared in three categories across the data as follows, according to who initiated the provision of the account and who ultimately provided it: (1) Other-initiated, self-provided—account following a direct solicitation by the teacher or peer (58%); (2) Other-initiated, other-provided account—child accounts for the peers’ behavior or work following a solicitation by the teacher (23%); and (3) Self-initiated, self-provided account—child accounts for their behavior without a direct solicitation but within the dialogue with the teacher (19%). Self-initiated, other-provided accounts were not found in the dataset. This type of account is defined by a sequence of turns where a speaker requests an explanation for their own behavior, and an interlocutor provides it. This is an unusual turn configuration, and as far as acknowledged, there is no documentation of such an account in the CA literature (Buttny, 1987). Within each type of account, we can identify four different modes of participation (see Fig. 2) and further understand how children gradually change the ways they position others and themselves in the group.

The proposed modes of participation are described considering the relationship among speakers, the position of the focus child (whose diary is being discussed) in the dialogue, and the constructions within the interaction itself. We propose that guided participation is the situation where interaction opportunities are created for the focus child; he or she is requested to join or respond to an action. In this case, although the child is given the possibility to take part in the process, their position is defined by another person in the group. On the opposite end, agentic participation is defined as the situation when the focus child is actively establishing the parameters of their participation in the conversation, and their turns in the sequence organization contribute to the continuation or deviation of the conversation. In between these two opposite modes we have a range of variable degrees that is dependent on the outcome of the interaction itself. Participation can lead to more constraining interactions, focusing on specific topics or narrowing the group to a dyadic interaction. Alternatively, participation can expand the conversation, bring new aspects to the topic, or include multiple points of view.

From a pedagogical point of view, the methodology of the Idea Diary privileged children’s perspectives and voices during the sharing circle, placing children in the center of the meaning-making process with epistemic authority over what is being discussed. The teacher’s flexibility was important in creating such a dynamic. This result is particularly relevant as it indicates the link between the teacher’s approach and children’s enhanced participation and will be addressed thoroughly in a different publication.

### Other-Initiated, Self-provided Accounts

The accounts in this category were related to the identity of objects, situations, and contents produced by the child. The sharing circles in the Idea Diary usually started with children showing their entries in the diary—drawings, collages, and writings that represented their experiences, learned contents, or interests in the past days. These accounts appeared immediately after two types of requests which are similar to those identified in previous works focusing on small-group discussions (Willemsen et al., 2018); (1) requests formatted as open invitations projecting descriptive answers, such as “what do you have here” and “what is this”; and (2) requests as open invitations projecting specific types of response, for example, “why have you done x”, which inherently targeted the child’s perceptions of their own experience.

**Fig. 3:**
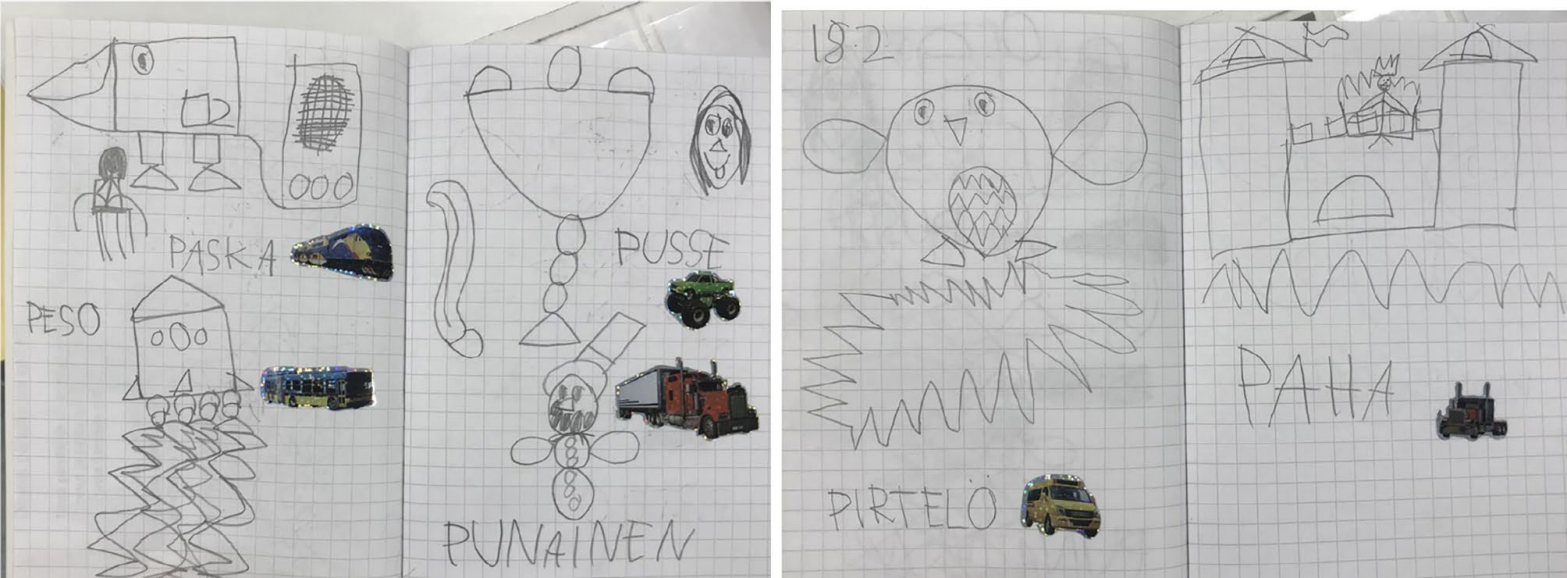
Child 2’s idea diary—Paska, peso, pusse, punainen (Sharing circle 1)

**Fig. 4:**
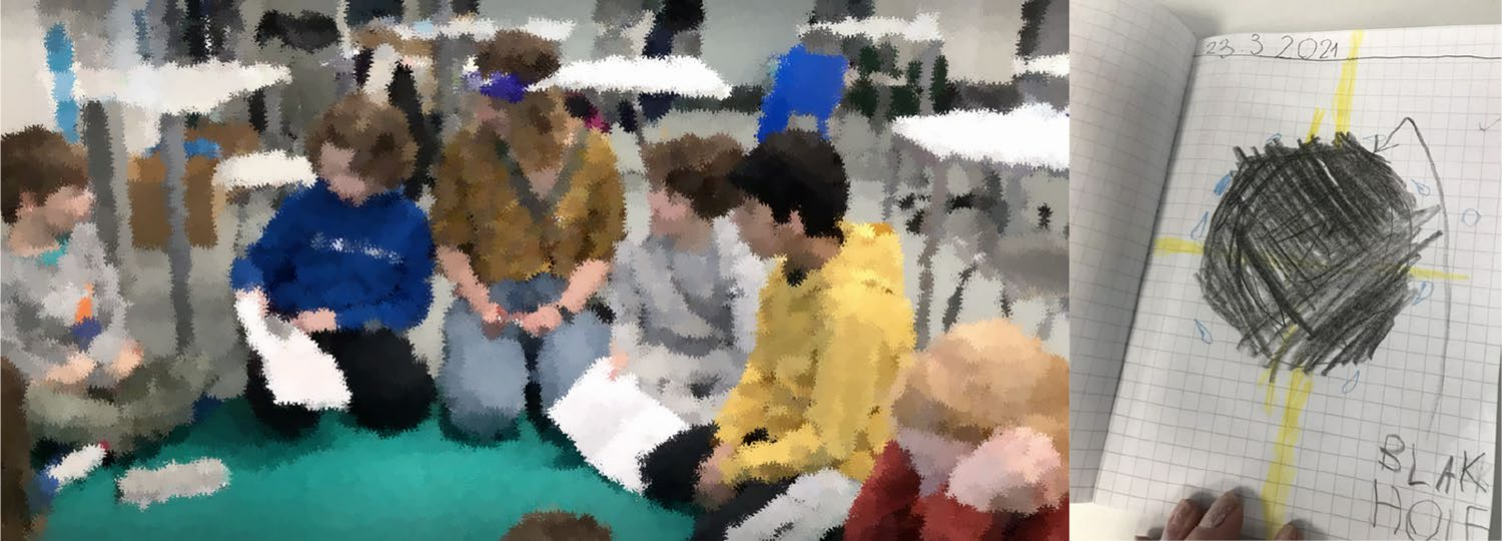
Child 3’s Diary—The blackhole (Sharing circle 6)

When producing other-initiated, self-provided accounts, children positioned themselves as part of the group by choosing whether and what to tell about the content represented in their diaries. Although the participation in these cases was guided (i.e., the teacher is requesting explanations, inviting children to join the sharing process) and constrained by the structure of a *wh*-questions, children made use of their position to explicate their thinking process, or to exert control over how they will conduct the sharing process according to their intentions. To illustrate, we present examples from the very first sharing circle (see Excerpt 1, Fig. 3) and sharing circle 6 (Excerpt 2, Fig. 4).

**Excerpt 1:**
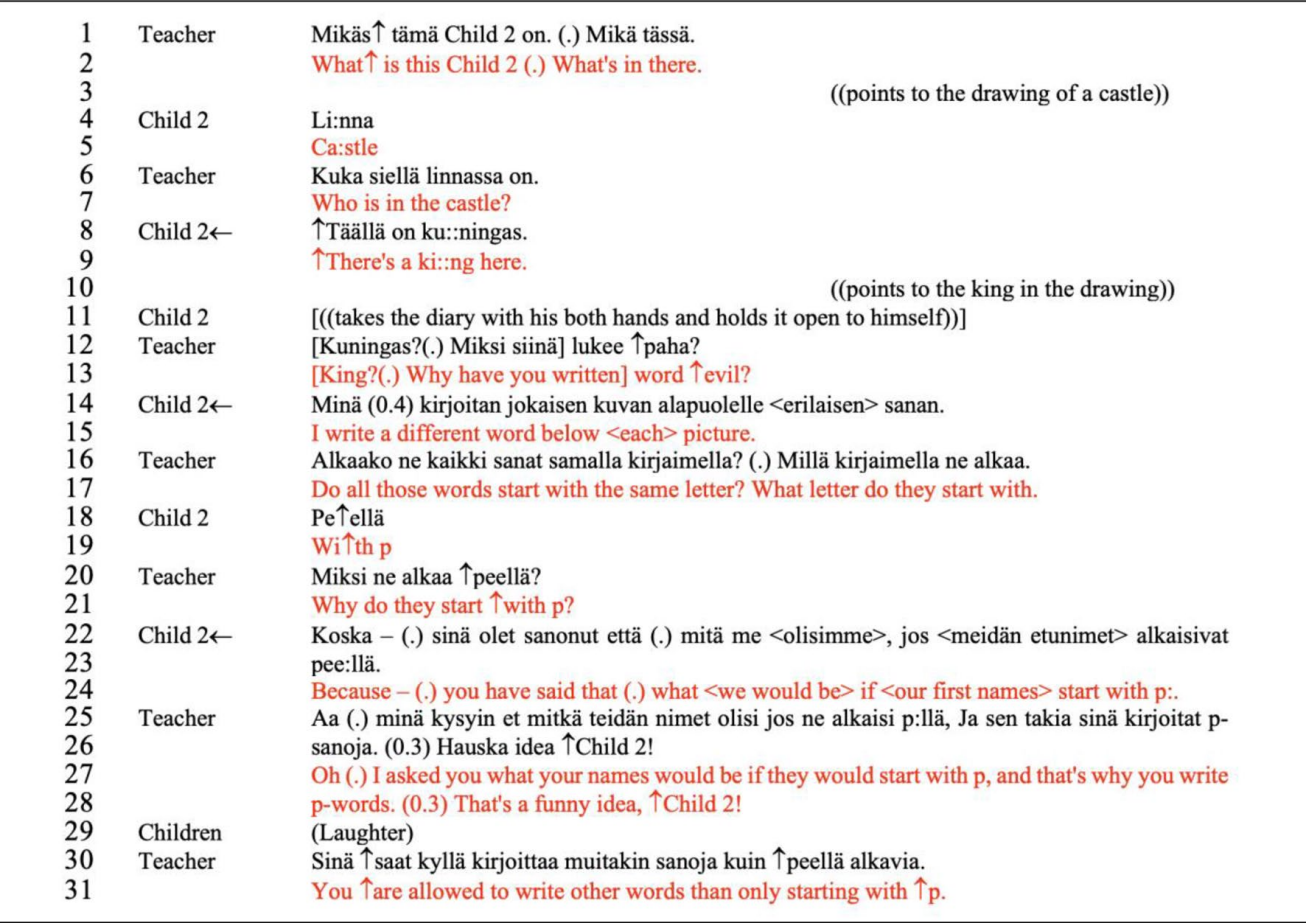
The letter “p”, like the pirate (Sharing circle 1, minute 8′ 25″–9′ 12″). Note: In the Finnish language, questions are signalled by a specific grammatical structure (use of the extension ko/kö at the end of the verb) or by using specific question words (e.g., missä, mihin, miten) with no necessary different intonation. Also, the Finnish language requires the pronunciation of all the letters, which naturally makes the continuation of the sound in words with double vowels or consonants; a situation that may influence the interpretation of this dialogue by non-Finnish speakers

**Excerpt 2:**
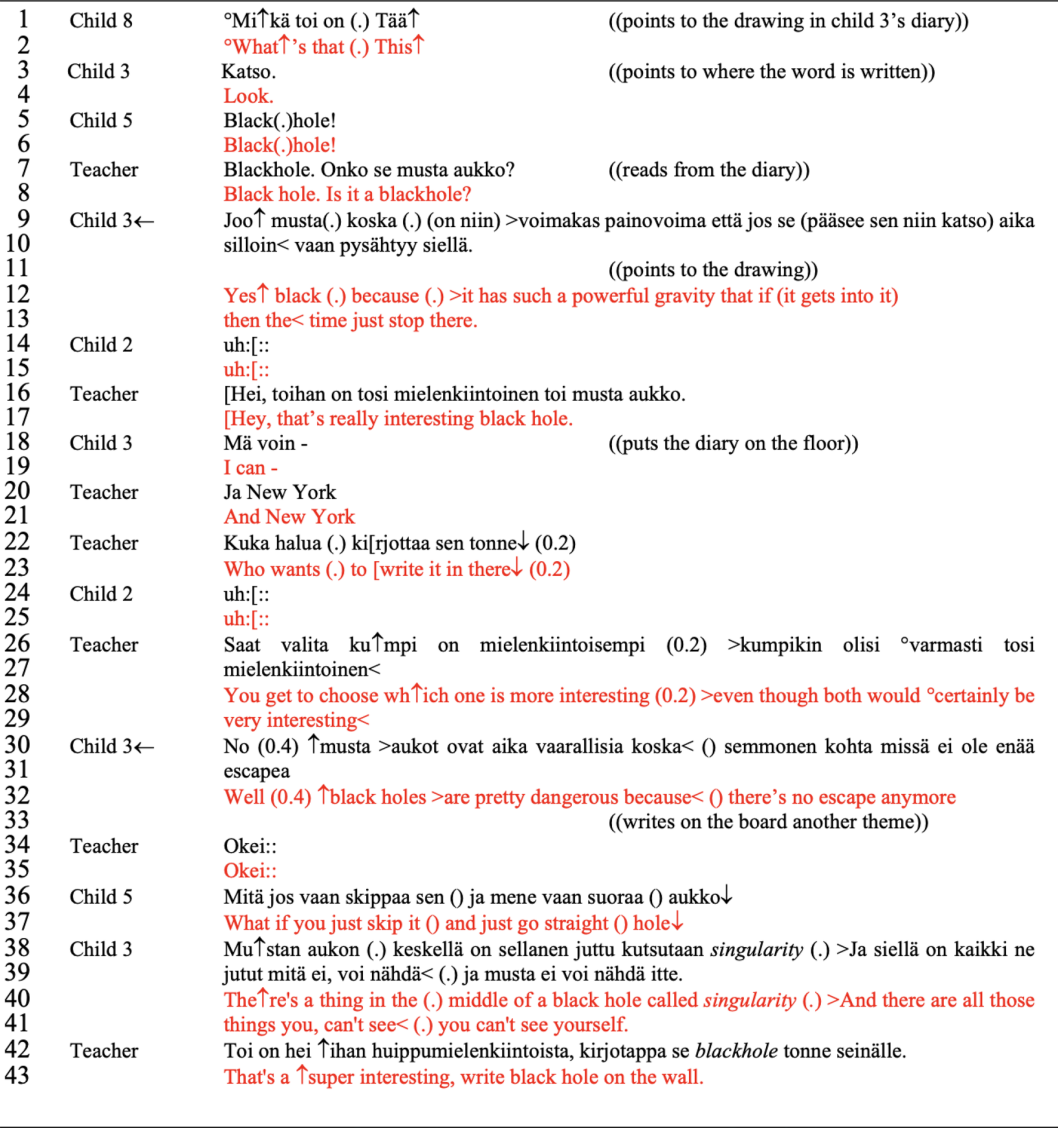
My knowledge about the blackholes (Sharing circle 6, minute 11′13’’ – 12′45’’)

In excerpt 1*—The letter “p” like the pirate*, we see an example in which open invitations projecting descriptive explanations (Willemsen et al., 2018) support the child to share not only the content but the reasoning (the reflective process of why they have noted something in a particular way) when registering ideas in the diary. The teacher identifies that child 2 assigns a random word that starts with the letter “p” to each of his drawings, which creates specific patterns of registering contents in the idea diary (see Fig. 3).

The teacher was particularly interested in the context behind the use of the word ‘evil’ (*paha*) and requests an account in line 12, which is then immediately provided by child 2; ‘I write a word below each picture. The child does not provide a direct explanation of how the word evil was thought about, but rather suggests in his turn that the word is random. This account is structured without the use of common explanatory conjunction (e.g., because) or reflective verbs (e.g. I think) featured in this type of adjacency pair. The account offers fragments of the information that was requested by the teacher (i.e., why something happened), demanding the interlocutor to interpret that the explanation is contained in the sentence “I write a word below each picture”. The dialogue continues focusing on the pattern established using the letter ‘p’ in all the words (lines 16 and 20), prompting a reflective process. In the following turn (line 22), child 2 provides the reasoning on how he engages with the construction of the diary and interestingly reveals the connection he made between the storytelling (*The pirate of words,* which tells the story of a pirate that collects words with the letter P) used to introduce the idea of using the diary and the actual construction of his diary. Once again, we see that the explanations provided by child 2 are elaborated as single words or short sentences bringing pieces of information (e.g., turns in lines 20–22), findings similar to other studies of young autistic children’s conversational moves (Bottema-Beutel, 2021; Ochs et al., 2004; Paul et al., 2009). From the perspective of the framework proposed in the Idea Diary (Muniz, 2015), participation and achievement are signaled in the prompt response and the adequacy of turn in responding to the teacher’s request for an account (i.e., the turn is “type fitted” to the sequence). The entire conversational structure and the consistent adjacency pairs formed by the child and the teacher reveal that child 2 has awareness of the processes involved in the construction of his idea diary and takes the opportunity of the small-group discussion to share his ideas. Participation, although guided, is assured both through engagement in the activity and the maintenance of communication in the small-group discussion.

Additionally, the constant use of the letter ‘p’ could be a sign of repetitive interest commonly associated with autism (APA, 2013; Bottema-Beutel et al., 2015), and our data manifests in a manner that supports the construction of the diary. The phenomenon of linguistic repetitiveness in autism is not limited to echolalic utterances (Dobbinson et al., 1998); it can also manifest in the repetition of a specific structure or a particular topic in the dialogue. For our analysis, it is relevant to consider that accounting for such patterns of behaviors creates an understanding of the child’s reasoning and execution of the diary, revealing the child’s reflective process about their work. The repetition is also a source of engagement in the Diary activity; it creates the regularity, consistency, and familiarity that autistic children seek in routines. Furthermore, considering the diary as a tool to express one’s ideas, a parallel can be made to the role of repetitions in autistic communications. Similar to the palilalia (repeats of one’s own prior talk) identified in autistic dialogues (Stribling et al., 2007), this child brings the reference of the letter ‘*p*’as a repetition of his own ‘turn’, creating a continuation for its engagement.

Another example of other initiated, self-provided accounts starts with a request from peers. In excerpt 2 (Excerpt 2)—*My knowledge about the blackholes* displayed below*,* child 8 inquired about specific content in child 3’s diary (line 1, see Fig. 4).

The request was followed by two interesting actions. First, child 3 in line 3 doesn’t offer a direct verbal explanation, but rather indicates where the peer can find the answer to his question. The lexical item ‘katso[look]’ expressed simultaneously with a deictic gesture (e.g., pointing) within a situation where the peer who uttered the question is already visually engaged is interpreted not as a directive to joint attention (which is already secured), but as an embodied supportive turn that mediates turn taking and projects an upcoming action (Bottema-Beutel et al., 2022). Beyond supporting the embodied engagement to the activity in place (i.e., encouraging peers to lean forward and continue looking at the diary), this turn in the dialogue shows that child 3 is expecting that child 8, like the rest of the group, can identify the picture since he has written the information right next to it (see Fig. 4). This is a clear example that child 3 has an idea of what other children should be able to do and organizes his materials around the expectation that other children can read. The following turn, expressed by child 5, answers child 8’s request for an account and confirms the embodied directive given by child 3. From a pedagogical point of view, child 3 is incorporating written language into his strategies to communicate thoughts and ideas, which is new behavior for any of the children in this group, and an important learning event for this child.

Second, this excerpt also illustrates the importance of recognizing each child’s epistemic authority (and their perception of others’ epistemic access) regarding their diary entries as part of supporting their participation and agency. When child 5 provides a possible explanation for 'black hole', the teacher reformulates the question (requests confirmation), pushing for a verbal confirmation (line 7), and by doing so, places the epistemic authority back on the child who made the drawing. This process prompts an account of why the drawing was made, informing the group about the topic, content, and experience in a broader way (see Excerpt 2, first arrow). The situation created by this sequence positions child 3 in the center of the learning dynamic; his experience, interests, and knowledge are recognized as relevant to the group and the learning process in place. Moreover, this context supports autonomous and intentional participation, which are further connected to developing a sense of agency as the understanding or awareness of the consequences of one’s actions in a certain event (Gallagher, 2007). Child 3 prepares the material by considering the sharing circle and the interaction with his peers; he not only writes the name of the figure (which could be a self-oriented behavior), but he uses the sign of the arrow to further evidence (transmit the information) of what he has drawn. The process of sharing specific knowledge is intentional; even further, he expects that others will recognize it and assures that peers and the teacher understand why he has drawn a “black hole” when accounting for the drawing on line 12.

### Other-Initiated, Other-Provided Account

In this category, children accounted for others’ behaviors or works and often portrayed children’s recalling events and reflecting on what seemed relevant to them about others’ productions. These types of accounts were usually offered as ‘candidate’ explanations that were up for negotiation. This framing is critical, because interlocutors do not usually have epistemic access to the reasons guiding their interaction partners’ behavior, and therefore cannot authoritatively provide accounts for their conduct. When children provided candidate explanations for their classmates’ conduct, this offered opportunities for interaction about characters and events on a meta-level, as well as explanations about the process of registering and sharing their ideas in the diary. Participation in these situations was still guided by the teacher’s or peers’ requests for accounts, but with fewer constraints of direct *wh*-requests, children’s participation could be expanded allowing the expression of their personal preferences, feelings, and opinions important to them. Resembling what happens when children are reading books together (Gosen et al., 2013; Rogoff, 1990), the conversations about the contents in the idea diary provided participation in cognitively challenging talk, and the extension of children’s knowledge related to the topics, concepts, and situations presented in the diaries.

**Fig. 5:**
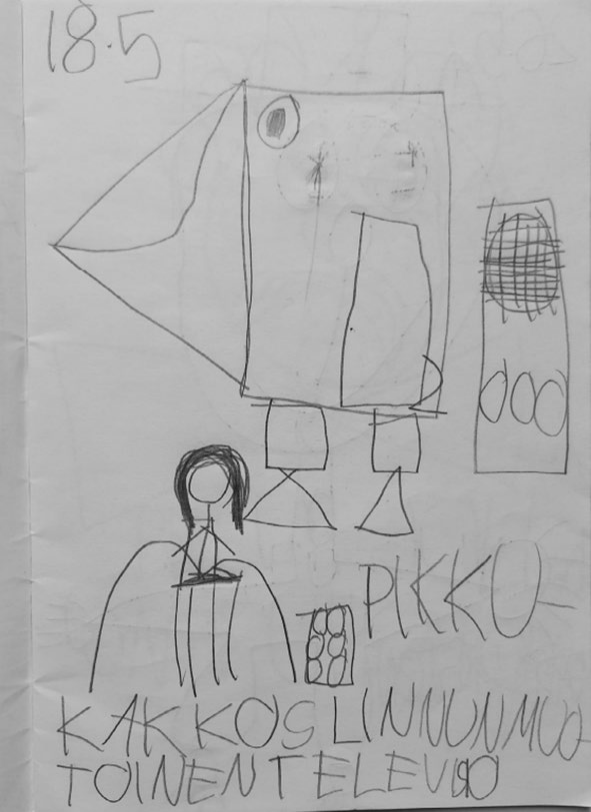
Child 2’ drawing

In excerpt 3—*Such a long word!* we see an example of an other-initiated, other-provided account that provides a child’s perception of the competencies of the peer. The account doesn’t change the configuration of children’s participation, but it discloses the understanding that children may have of each other within the group. In this small-group discussion, children are starting to explore child 2’s diary. Child 2 has drawn a figure that he invented (see Fig. 5) and the teacher is interested in the meaning behind the rather distinct drawing.

**Excerpt 3:**
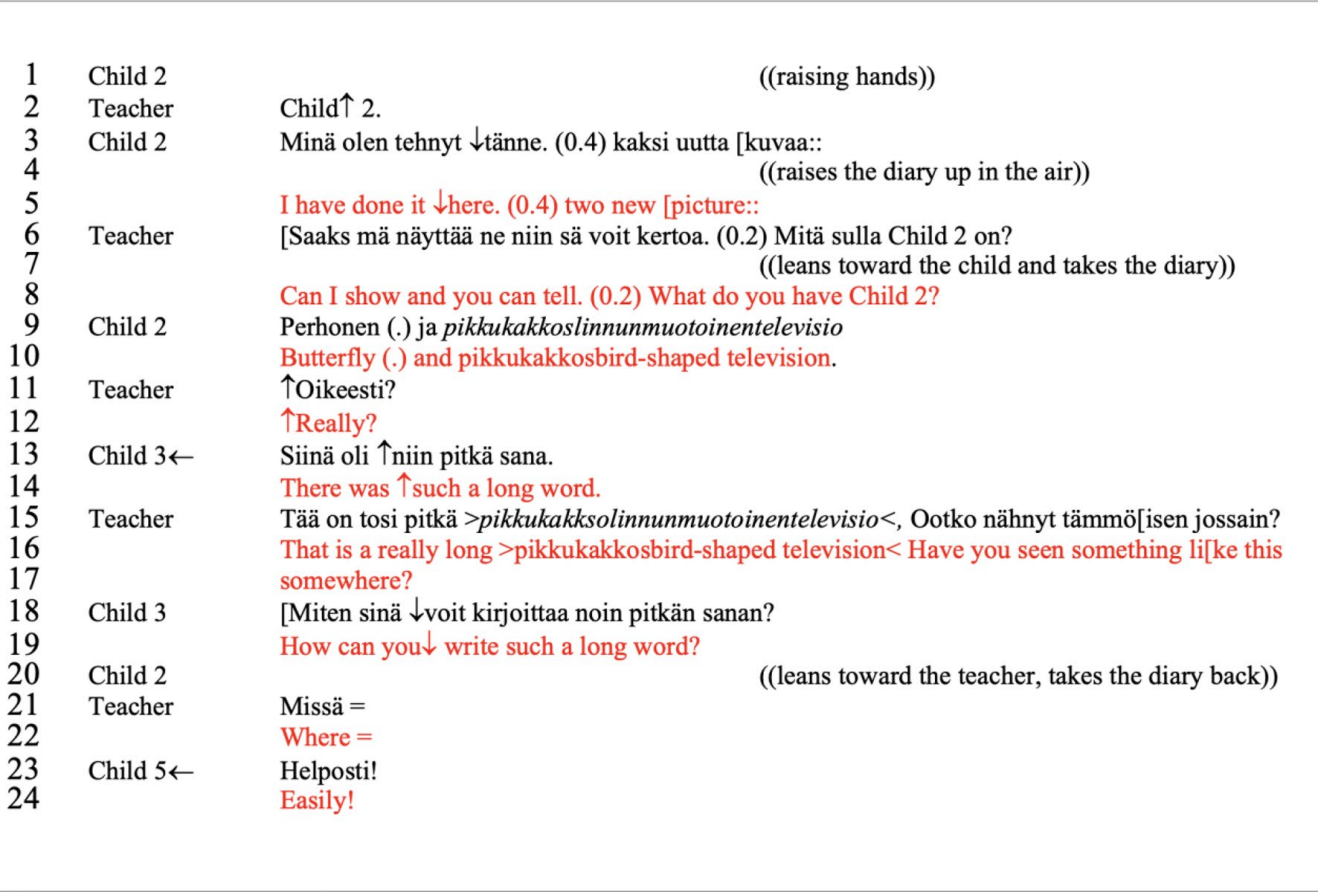
Such a long word! (Sharing circle 10, minute 2′ 45″–3′ 57″)

The conversation starts with child 2’s initiative in requesting the floor by raising his hands during a pause in the previous sequences (line 1). The teacher acknowledges the request, and invites him to speak by uttering his name with high intonation (line 2) and focusing her gaze on the child. Child 2 takes the turn and directs the attention of the group toward his diary “I have done here two new drawings”, pre-setting the subsequent invitation for the group to explore child 2’s diary, which is completed with the bodily action in line 4. This sequence shows that child 2 understands the conversational structure of the sharing circle and is acting accordingly to what is expected in a small-group discussion—identify a moment to change turns, request a turn, and propose action. Although the turns in this sequence are not clearly articulated in verbal utterances, the bodily actions perform the invitation for others’ to participate.

In the following sequence (lines 6–9), the teacher initiated a new adjacency pair (type offer-acceptance; Stivers, 2012) offering to assist child 2 in showing the diary (line 6 “Can I show and you tell”) and scaffolded the following turns by elaborating on a more direct question to guide child 2’s presentation (continuation of line 6 “what do you have there, child 2?”). With this combination of utterances, the teacher sets the ground for exploring child 2’s diary; not only does child 2 have the floor to present his drawings, but he also is supported on how to begin the explanation of his doings (line 9). Breaking down complex interactional settings into more direct and simple ones supports the continuation of the dialogue (Bottema-Beutel, 2017; Rendle-Short, 2014; Sterponi & Shankey, 2014), thus increasing engagement and participation. This type of interactional scaffolding is commonly identified in conversations with autistic people (Rendle-Short, 2014) and it occurred several times in our data set.

In the continuation of this sequence, we see that both teacher and children are amazed and even hesitant with the word “*pikkukakkoslinnunmuotoinentelevisio”*. In line 11, the teacher asks for confirmation if this is correct, and in line 13 (marked by the arrow) child 13 makes a remark about the length of the word that is interpreted as a possible account of why the teacher needs to check. Continuing with the line of questioning, the teacher and child 3 reaffirm their curiosity and doubt about how child 2 created the figure and the word, both imposing a question. However, what happens next is that in line 23 child 5 will account for the questions (other-initiated, other-provided account), ending the questioning and validating child 2’s ability to create such a figure. The account changes the content of the dialogue, promoting inquiry, not about the validity of the material produced but about the exploration of the meanings it entails.

### Self-Initiated, Self-Provided

This type of account appeared formulated as spontaneous explanations about children’s own (within the dyad child-teacher) or others’ work (interjecting an ongoing dialogue between the teacher and another child). The accounts either added new information to the ongoing discussion or added remarks of personal opinions about peers’ work. Expansions precede, intervene in, or follow the base of the sequence, and can indicate stance, managing affiliation or alignment, or dealing with issues of intersubjectivity (Stivers, 2012). Structuring spontaneous initiations can be a challenge for autistic people due to the demand of considering various factors involved in social dynamics (Colombi et al., 2009; Fujiki & Brinton, 2009; Koegel et al., 2003). However, conversation analysis has revealed the communication competencies of autistic people on different occasions (Conn et al., 2018; Rendle-Short, 2014; Stiegler, 2007) showing that people on the spectrum may deploy unconventional means to perform the same pragmatic function in the dialogue as conventional conversational structures.

In our data, the self-initiated, self-provided accounts often followed confirmation statements or were structured within a single turn at talk. The latter occurs when a speaker perceives that something in their utterance requires explanation, before such an explanation is requested (Heritage, 1988). The first example of a self-initiated, self-provided account can be found in excerpt 2, presented previously. On lines 29 and 30 (emphasized with the second arrow), the teacher reaffirms the relevance of the turn presented by child 3 and immediately proposes that he chooses the topic of next week’s classwork (which is part of the methodology of the Idea Diary). Prior research has already shown that formulating proposals so they reflect what the child is already doing increases the likelihood that the child will continue engaging with the task at hand (Bottema-Beutel et al., 2018). Child 3 reflects on the proposal (identified by the time between the first response ‘well’ and the account that follows) and accounts for his decision not to choose the topic of the black hole (the topic that child 3 writes on the board, just after the dialogue presented in excerpt 2). Although the formulation of this account is not clearly structured as a reflection (e.g., ‘I will choose this because I am afraid of choosing that’), child 3 anticipates that a justification is necessary (provides the account before writing the word on the board) for what he is about to do next. The account is configured around the fundamental motivation behind the child’s decision, and gives evidence that the child orients to how his interaction partners make sense of his conduct.

**Fig. 6:**
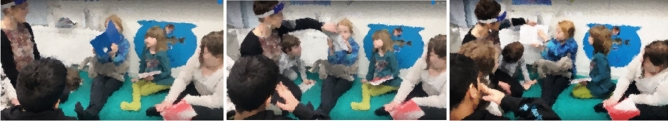
Child 2 presents the idea diary according to his strategy (Sharing circle 1). The series of pictures are representative of lines: 12, 22, and 33, respectively

In a slightly different manner, In excerpt 4—*Establishing the dynamic of the presentation*, child 2 explains (line 13) his desired way of participating after the teacher proposes a different way of participating (line 1). The child anticipates the rules of the group dynamic and positions himself as the ‘one ahead of the process of sharing’, conducting the presentation. In this interaction, it is important to highlight how the teacher accepts the child’s explanation and flexibly follows the child’s proposal for the presentation (shown in line 27). To illustrate the dialogue and the embodied actions that support this interpretation, we present excerpt 4 and Fig. 6.

**Excerpt 4:**
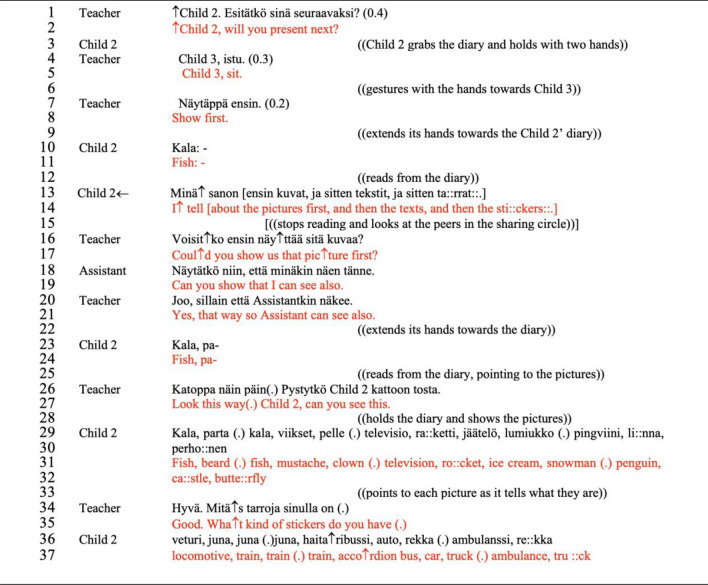
Establishing the dynamic of the presentation (Sharing circle 1, minute 6′59’’ – 8′10’’)

Overall, self-initiated accounts offer evidence that autistic children anticipate their interlocutors’ perception of their conduct and,, different from what previous studies have found (Angus et al., 2015), are capable of providing the answer to an imagined request for explanation. From the conversational point of view, self-initiated, self-provided accounts were used to inform the group of something relevant about the topic, personalizing the experience and revealing motivational stances surrounding the construction of the Diary.

Looking from the perspective of children’s participation, self-initiated accounts in this study supported children to experience the construction of continuous dialogues more autonomously and spontaneously, acting, expressing ideas and perceptions, and contributing to the small-group discussion. Participation promoted by self-initiated accounts served the purpose of entering and influencing others’ sense-making processes, focalizing on a specific topic of the conversation, and effectively regulating other peers’ and the teacher’s behaviors. It is within the self-initiated, self-provided accounts that children are revealing the meaning-making processes, providing the opportunity for the teacher to understand their world of significance and from that standpoint further work with the child.

## Discussion

In the present study, we examined interactional sequences that contained accounts provided by autistic children while participating in discussions about the Idea Diary. The study elaborated on two guiding research questions: What types of accounts do autistic children provide when talking about their work and ideas? And how do accounts emerge and support participation in small-group discussions?

Regarding the types of accounts, our analysis revealed that accounts in conversations with autistic children emerge in varied sequences and serve different purposes in a dialogue, exhibiting similar complexity as expected in the analysis of explanatory acts in social interactions among neurotypical children and adults (Heritage, 1988). More precisely, in our study, three categories of accounts were identified in children’s dialogues during sharing circles. By analyzing the embedded accounts' sequences, we identified a particular mode of participation supported by the activity with the Idea Diary. All the modes of participation were considered relevant for children’s engagement in the small-group discussion; thus, hierarchical discrimination between them was not established, and all deserve further scrutiny in future research.

Accounts were initiated by others (teacher and peers—other initiated) and by children themselves (self-initiated). In both situations, we understand that providing acceptable reasons for one’s actions demands that children learn what kinds of evidence and what kind of explanation others will consider adequate on a particular occasion (Goetz & Shatz, 1999); they must understand the structure of the social dynamic in place and have an idea on how they can situate themselves in the dynamic. The development and the structure of explanations have been extensively studied in the context of peer conflict (e.g., Blum-Kulka et al., 2010; Dunn & Brown, 1993), and research has shown that children learn how to negotiate, justify, and explain their behaviors, actions, and beliefs from a very early age. In such studies, learning how to produce explanations in different conversational contexts is identified by the use of using causal connectives (e.g., *because*, *so*), or mental state verbs (e.g., *I think*, *I know*), and the explanatory act is interpreted as a sign of the child’s understanding of others' mental states (a reference to the Theory of Mind, Matthews & Goldberg, 2018), and acting upon this understanding. Although in our study, children did not make use of the most common linguistic forms of responding to requests for explanation, such as causal connectives or mental state verbs, they provided the reasons justifying certain content and anticipated responses (e.g., In excerpt 2—*My knowledge about the blackholes*), showing understanding of the conversational structure and even challenging the idea that autistic children are not capable of practical reasoning regarding what interaction partners may need to know about their own and others’ behavior, as assumed in ToM framework (see also Henderson, 2019 for further insights on this point).

In our study, children produced accounts for their actions, beliefs, and behaviors building on the conversation (e.g., excerpt 1), positioning themselves and influencing the direction of the conversation (e.g., excerpt 3), and establishing boundaries for the interaction (e.g., excerpt 4), offering a glimpse of autistic sense-making and participatory sense-making in the process (Di Paolo et al., 2018). The interactions presented here are examples of, as defined by enactive theories of social cognition (De Jaegher & Di Paolo, 2007, 2013), coupling between two or more autonomous agents, co-regulated by the interactors while sustaining the balance between maintaining individual identity and exchanging with the world (De Jaegher & Di Paolo, 2013). Children participating in interactions structured by the Idea Diary find it relevant to share their ideas and engage other students in their learning experiences. They see themselves as part of the group (members of the group as constitutive of their identity); thus the sharing experience is materialized because within the interaction children find it equally important to share their thoughts and listen to others’ ideas.

Our study offers evidence of spontaneous interference of others' perception within dialog (self-initiated, self-provided accounts). The occurrence of such types of accounts in specific moments of the data collection suggests that although spontaneous communication can be challenging for an autistic child (Fujiki & Brinton, 2009), interactions that are structured yet flexible (in our case, the structure of the Idea Diary sharing circles) may help autistic children be more comfortable negotiating interactions. Our analysis did not identify what aspects of the interactions supported children to express spontaneous accounts. One possibility is that the repetitive configuration of sharing circles (i.e., the protocol to which the teacher conducts the sessions) could make the conversation structure predictable and, therefore, more manageable for autistic children. Knowing when the diary happens (class routine), how it starts (e.g., always starting by asking who wants to share and going around in the circle), and what the conversation patterns created by the teacher and peers will be (e.g., inviting children to tell what they have done and why) informs children on how to behave and prepare for the interaction. At the same time, the Idea Diary allows flexibility and for children to speak freely, with no right or wrong answers (Muniz, 2020). Children are invited to talk about themselves and their points of view on important topics. This combination can grant the gradual building of confidence necessary for constructing a dialogue.

Another interesting configuration is the one created with other-initiated, self-provided and other-provided accounts in situations where children discuss peers’ Idea Diaries. In such moments, we can notice how children gradually take interest in others’ experiences and ideas, engaging in various topics. Other-initiated accounts show the moments when children engage with what is being presented, entering their peer’s subjective world (i.e., ideas, experiences, feelings, and desires). This context prompts actions in conversation, such as requesting clarifications. In our study, possible explanations for what is being presented or discussed are provided by the child responsible for the Diary in discussion (self-provided accounts) and other peers in the small group (other-provided accounts). Through this process, children are building up joint narratives and (re)signifying the experiences that are being shared. These elements are juxtaposed in our reflections; they belong to a complicated mesh of intersubjective dynamics that constitute everyday social interaction (Gonzaléz-Rey, 2018).

Regarding how accounts supported participation, we have found in our collection of accounts that children were competent communicators of their ideas, interests, and knowledge. The accounts in the present study showed how autistic children explicate their thinking process, often taking over the control of how they will conduct the sharing process according to their intentions. Our findings also revealed a great deal of active involvement and agency in the construction of small group discussions, which is understood to be related to holding epistemic authority over the knowledge involved in such types of interactions. Holding epistemic authority on conversation topics seems essential in enhancing children’s participation, even if the group discussion structure is under the teacher's guidance. Thus, children should be further encouraged to exercise small-group conversation where the epistemic authority lies with them.

The Idea Diary also supported children in expanding their reference and understanding how they are active participants in the knowledge construction process. Children in this study were interested and engaged in sharing their experiences, knowledge, and ideas, including experiences outside of school. Experiences from other social contexts than school opened new avenues of discussion and encouraged children to explore other sources of information, such as the internet, books, and conversations with parents. The conversations about the contents in the idea diary provided participation in cognitively challenging talk, and the extension of children’s knowledge related to the topics, concepts, and situations presented in the diaries.

In our study, the teacher used an open and flexible approach for soliciting and responding to children’s contributions. Through analysis of different sequences, we captured the teacher supporting reflection (excerpt 1), validating children’s interests (excerpt 2), and encouraging children to participate without establishing one way in which this participation should happen (excerpt 4). The accounts were often initiated by the teacher and on different occasions, scaffolding turns were used to support children’s explanations. The teacher provided an engaged, sensitive interaction that fits well with the description of a ‘*letting be* attitude’ conceptualized by De Jaegher (2020). The idea of ‘*letting be’* translates into finding the necessary “balance between the ongoing becomings of both known and knower” (De Jaegher, 2020, p.10), and conveys the idea that interactions have a life of their own and depend on the maintenance of the self-organization of those involved. In the context of this study, the teacher (often in the role of the knower) as the one that is responsible for organizing and restructuring the sharing session provided space and opportunity for children to be in the relationship and to be themselves. This *letting-be* attitude is not an exclusive product of the Idea Diary dynamic, and as pointed out by De Jeagher (2020) has rather been present in many previous studies (Bottema-Beutel, 2017; Holt &Yuill, 2017; Park, 2012), showing that a key feature of participation between autistic and non-autistic people is sensitivity and openness.

### Final Considerations

The educational value of exploring conversation among autistic children is, we believe, self-evident. The first point of contact between educators and peers in the school is through interaction, then a detailed analysis of that interaction is bound to be a useful undertaking, especially considering that conversation with autistic children can often be challenging for non-autistic people who are not experienced in appropriately adapting their conversational repertoires. The findings presented in this article indicate how autistic children can build on their understanding of social dynamics when the epistemic authority necessary for knowledge construction belongs to them, showing issues of broader significance within educational research in autism. In particular, the shift in emphasis away from the autistic person as the source of trouble in interaction, and re-focus on the interaction itself can allow the possibility of modifying the interaction in such a way as to enhance the communicative exchange for all participants. Therefore, the present study contributes to the long-standing discussion on the importance of supporting social interactions, group learning, and peer collaboration in educational contexts for autistic children (Fantasia et al., 2014), arguing alongside others that these children are socially competent (Caldwell, 2006) and that deficit-based practices only reinforce the idea that non-autistic interactions are more valuable. The present study indicates that pedagogical approaches that use learning strategies in which autistic children lead the activity and hold the epistemic authority of knowledge construction support autistic children’s interactions, evidencing interactive dynamics that feature accountability, mutual engagement, and agency.

Moreover, the study also offers interesting inputs on using CA in dialogues among Finnish autistic children for two reasons. First, it addresses a phenomenon (small group interactions among autistic children) that has rarely been explored with the use of CA. For example, according to the Finnish Conversation Analysis Archive (https://metashare.csc.fi/)—a collection of recordings of Finnish speech in interaction under the domain of Helsinki University and widely used by CA researchers in Finland, there aren’t any samples of small-group interactions among Finnish autistic children. Second, the study explores the possibilities and challenges of applying CA conventions created for English speakers in a completely different language, such as Finnish, pointing out the need to further develop CA methods and conventions in languages other than English. In this study, we identified the need to adjust Jefferson's (1986) conventions. For example, different from English, Finnish relies on specific grammatical structures (kö/ko) instead of intonations to create certain types of questions, or it demands the pronunciation of every letter in the word, which can sound like a prolongation of the word. Thus, the translation work must be culturally sensitive.

### Limitations and Considerations for Future Studies

Children participating in this study were not diagnosed or evaluated systematically by any of the researchers, evaluations were instead carried out by the school personnel. Specificities of the spectrum such as parameters of language development and social skills were not used to determine the inclusion criteria or baseline for this study. Therefore, it is important to consider individual variations and recognize that case study analysis is designed to explore a given phenomenon in-depth, and not to generalize findings beyond the data. While keeping in mind the qualitative and small-scale character of the present study, we reflect upon two important considerations for future studies – the role of teacher’s communication strategies for supporting group learning among autistic children, and the further exploration of the dynamic system created among autistic children (peer-to-peer). Teachers’ communication strategies can vary significantly even within the same activities (Willemsen et al., 2018). However, in this study, we noticed that the teacher kept a consistent way of interacting with children that involved flexibility and acceptance of the ways by which they decided to engage, which will be presented in a different publication. How many of the advances children made concerning their participation are due to the flexible approach of the teacher? This perspective will be explored in the future by using the Enactive theories of intersubjectivity (Di Paolo et al., 2018) to analyze the dynamics created by this communication strategy.

In conclusion, the present article reports an interesting case of an alternative student-centered pedagogical tool for working with children receiving special support. The study evidence important communicative competencies of autistic children that support their learning and participation in small-group discussions in school activities. Namely, our findings showed that autistic children engaged in constructing explanations for their and others’ conduct, which illustrates their sensitivity in attending to the practical needs of their interaction partners in order to maintain intersubjectivity while propelling the interaction forward.

### Supplementary Information

Below is the link to the electronic supplementary material.

Electronic supplementary material 1 (DOCX 113 kb)

## Footnotes

1 Information on the project can be found on the webpage: https://research.tuni.fi/ecepp/projects/

## References

[CR1] Ainscow M (2016). Diversity and equity: A global education challenge. Journal of Educational Studies.

[CR2] Ainscow M, Messiou K (2021). Inclusive Inquiry: An innovative approach to promote inclusion in schools. Revista Latinoamericana De Educación Inclusiva.

[CR3] American Psychiatric Association (2013). Diagnostic and statistical manual of mental disorders.

[CR4] Angus DJ, Rosnay M, Lunenburg P, Meerum Terwogt M, Begeer S (2015). Limitations in social anticipation are independent of imaginative and Theory of Mind abilities in children with autism but not in typically developing children. Autism: The International Journal of Research and Practice.

[CR5] Antaki C (1994). Explaining and arguing: The social organisation of accounts.

[CR6] Antaki C, Fielding G, Antaki C (1981). Research on ordinary explanations. The psychology of ordinary explanations of social behaviour.

[CR7] Applebee AN, Langer JA, Nystrand M, Gamoran A (2003). Discussion-based approaches to developing understanding: Classroom instruction and student performance in middle and high school English. American Educational Research Journal.

[CR8] Black-Hawkins K, Florian L, Rouse M (2007). Achievement and inclusion in schools.

[CR9] Blum-Kulka S, Hamo M, Habib T (2010). Explanations in naturally occurring peer talk: Conversational emergence and function, thematic scope, and contribution to the development of discursive skills. First Language.

[CR10] Bottema-Beutel K, Louick R, White R (2015). Repetition, response mobilization, and face: Analysis of group interactions with a 19-year-old with Asperger syndrome. Journal of Communication Disorders.

[CR11] Bottema-Beutel K (2017). Glimpses into the blind spot: Social interaction and autism. Journal of Communication Disorders.

[CR12] Bottema-Beutel K, Lloyd B, Watson L, Yoder P (2018). Bidirectional influences of caregiver utterances and supported joint engagement in children with and without autism spectrum disorder. Autism Research.

[CR13] Bottema-Beutel K, Kapp SK, Lester JN, Sasson NJ, Hand BN (2021). Autism in Adulthood.

[CR14] Bottema-Beutel K, Crowley S, Kim SY (2022). Sequence organization of autistic children’s play with caregivers: Rethinking follow-in directives. Autism, The International Journal of Research and Practice.

[CR15] Chinn CA, Anderson RC, Waggoner MA (2001). Patterns of discourse in two kinds of literature discussion. Reading Research Quarterly.

[CR16] Colombi C, Liebal K, Tomasello M, Young G, Warnekem F, Rogers SJ (2009). Examining correlates of cooperation in autism, imitation, joint attention, and understanding intentions. Autism.

[CR17] Conn C, Lewis M, Matthews S (2018). An analysis of educational dialogue as support for learning young pupils with autism in mainstream schools. International Journal of Inclusive Education.

[CR18] De Jaegher H (2021). Seeing and inviting participation in autistic interactions. Transcultural Psychiatry.

[CR19] De Jaegher H, Di Paolo E (2007). Participatory sense-making: An enactive approach to social cognition. Phenomenology and the Cognitive Sciences.

[CR20] De Jaegher H, Di Paolo E (2013). Enactivism is not interactionism. Frontiers in Human Neuroscience.

[CR21] Di Paolo E, Cuffari E, De Jaegher H (2018). Linguistic bodies.

[CR22] Dobbinson S, Perkins MR, Boucher J (1998). Structural patterns in conversations with a woman who has autism. Journal of Communication Disorders.

[CR23] Dunn J, Brown J (1993). Early conversations about causality: Content, pragmatics and developmental change. British Journal of Developmental Psychology.

[CR24] EDUFI - Finnish National Board of Education. (2016). National core curriculum for basic education 2014.

[CR25] European Agency for Development in Special Needs Education. (2011). Participation in Inclusive Education: Framework for Developing Indicators. Odense, Denmark: European Agency for Development in Special Needs Education.

[CR26] European Agency for Special Needs and Inclusive Education. (2018). *Methodology report. Update 2018*. Retrieved from https://www.european-agency.org/sites/default/files/easie_methodology

[CR27] Fantasia V, De Jaegher H, Fasulo A (2014). We can work it out: An enactive look at cooperation. Frontiers in Psychology.

[CR28] Fasano RM, Perry LK, Zhang Y, Vitale L, Wang J, Song C, Messinger DS (2021). A granular perspective on inclusion: Objectively measured interactions of preschoolers with and without autism. Autism Research.

[CR29] Ferreira. J.M., Mäkinen. M., & Mäkihonko, M. (2022, accepted for publication). Equality, equity, and the child’s best interest as guiding principles in the implementation of the Finnish inclusive education system. In G. M. Kraemer, K. Wunder & L. B. Lopes *A educação das pessoas com deficiência: Desafios, Perspectivas e Possibilidades.* Apris Editora.

[CR30] Fujiki M, Brinton B, Schwartz RG (2009). Pragmatics and social communication in child language disorders. Handbook of child language disorders.

[CR31] Gallagher S (2007). The Natural Philosophy of Agency. Philosophy Compass.

[CR32] Goetz P, Shatz M (1999). When and how peers give reasons: Justifications in the talk of middle school children. Journal of Child Language.

[CR33] González Rey F (2018). Subjectivity and discourse: Complementary topics for a critical psychology. Culture & Psychology.

[CR34] Gosen MN, Berenst J, de Glopper K (2013). The interactional structure of explanations during shared reading at kindergarten. International Journal of Educational Research.

[CR35] Grosse G, Tomasello M (2012). Two-year-old children differentiate test questions from genuine questions. Journal of Child Language.

[CR36] Henderson GE (2019). Autistic children’s explanations of their own behavior: Evidence of other-attentiveness. Journal of Interactional Research in Communication Disorders.

[CR37] Heritage J, Antaki C (1988). Explanations as accounts: A conversational analytic perspective. Analysing everyday explanations: A case of methods.

[CR38] Heritage J, Sidnell J, Stivers T (2012). Epistemics in conversation. The handbook of conversation analysis.

[CR39] Hughes C, Kaplan L, Bernstein R, Boykin M, Reilly C, Brigham N, Cosgriff J, Heilingoetter J, Harvey M (2012). Increasing social interaction skills of secondary school students with autism and/or intellectual disability: A review of interventions. Research and Practice for Persons with Severe Disabilities.

[CR40] Hutchby I, Wooffitt R (1998). Conversation analysis: Principles, practices, and applications.

[CR41] Ingram J, Andrews N, Pitt A, Moschkovich JN, Wagner D, Bose A, Mendes JR, Schütte M (2018). Making student explanations relevant in whole class discussion. Language and communication in mathematics education.

[CR42] Jefferson G, Atkinson JM, Heritage J (1986). Transcript notation. Structures of social action studies in conversational analysis.

[CR43] Koegel LK, Camarata SM, Valdez-Menchaca M, Koegel RL (1998). Setting generalization of question-asking by children with autism. American Journal on Mental Retardation.

[CR44] Koegel LK, Carter C, Koegel R (2003). Teaching children with autism self-initiations as a pivotal response. Topics in Language Disorders.

[CR45] Koole T, Berenst J, Deen J, Hajer M, Koole T (2008). Pupil participation in plenary interaction. Interaction in two multicultural mathematics classrooms: Mechanisms of inclusion and exclusion.

[CR46] Levinson SC (1983). Pragmatics.

[CR47] Lima, M. E. M., Ferreira, J. M., & Vieira A. M. (2022). Criatividade nos registros feitos pelas crianças no Diário de Ideias . In L. Muniz, and J. Moran, *Diário de ideias: pilares teórico-metodológicos e experiências de implementação em escolas públicas.* Edufu, Editora Universitária.

[CR48] Locke J, Kasari C, Rotheram-Fuller M, Jacobs J (2013). Social network changes over the school year among elementary school-aged children with and without an autism spectrum disorder. School Mental Health.

[CR49] Maine F, Hofmann R (2016). Talking for meaning: The dialogic engagement of teachers and children in small group reading context. International Journal of Educational Research.

[CR50] Matthews NL, Goldberg WA (2018). Theory of mind in children with and without autism spectrum disorder: Associations with the sibling constellation. Autism.

[CR51] McCann J, Peppé S (2003). Prosody in autism spectrum disorder: A critical review. International Journal of Language and Communication Disorder.

[CR52] McKeown MG, Beck IL, Ronette GK, Blake RG (2009). Rethinking reading comprehension instruction: A comparison of instruction for strategies and content approaches. Reading Research Quarterly.

[CR53] Mercer N (2000). Words & minds: How we use language to think together.

[CR54] Muniz, L. S. (2015). *Aprendizagem criativa da leitura e da escrita e suas inter-relações com o desenvolvimento da subjetividade da criança* (publication n. 315) [Doctoral dissertation,Universidade de Brasília]. UNB Publishing. http://dx.doi.org/10.26512/2015.04.T.19015

[CR55] Muniz LS (2020). Diário de Ideas: Linhas de experiência.

[CR56] Muniz, L. S., & Mitjáns-Martinez, A. (Eds) (2019). *Aprendizagem Criativa da Leitura e da Escrita e Desenvolvimento: Princípios e Estratégias do Trabalho Pedagógico*. Apris

[CR57] Murphy PK, Wilkinson IAG, Soter AO, Hennessey MN, Alexander JF (2009). Examining the effects of classroom discussion on students’ comprehension of text: A meta-analysis. Journal of Educational Psychology.

[CR58] Myhill D (2006). Talk, talk, talk: Teaching and learning in whole class discourse. Research Papers in Education.

[CR59] Ochs E, Solomon O (2004). Introduction: Discourse and autism. Discourse Studies.

[CR60] Ochs E, Solomon O (2010). Autistic sociality. Ethos.

[CR61] Ochs E, Kremer-Sadlik T, Gainer Sirota K, Solomon O (2004). Autism and the social world: An anthropological perspective. Discourse Studies.

[CR62] OSF—Official Statistics of Finland. (2020). Special education [e-publication]. ISSN=1799-1617. 2020. Helsinki: Statistics Finland [referred: 14.2.2022]. Available: http://www.stat.fi/til/erop/2020/erop_2020_2021-06-08_tie_001_en.html

[CR63] Oliveira, A. M. C., & González Rey, F. (2018). Subjectivity Development in the Classroom: Learning Processes from a Cultural-Historical Standpoint. *The International Journal of Early Childhood Learning, 25*(2), 1–13. http://doi.org/10.18848/2327-7939/CGP/v25i02/1-13.

[CR64] O’Reilly M, Lester JN, Muskett T (2016). Discourse/conversation analysis and autism spectrum disorder. Journal of Autism Developmental Disorder.

[CR65] Paul R, Miles S, Cicchetti D, Sparrow S, Klin A, Volkmar F, Coflin M, Booker S (2004). Adaptive behavior in autism and developmental pervasive development disorder—Not otherwise specified: Microanalysis of scores on the Vineland Adaptive Behavior Scales. Journal of Autism and Developmental Disorders.

[CR66] Paul R, Orlovski SM, Marcinko HC, Volkmar F (2009). Conversational behaviors in youth with high-functioning ASD and Asperger syndrome. Journal of Autism and Developmental Disorders.

[CR67] Price-Williams D, Sabsay S (1979). Communicative competence among severely retarded persons. Semiotica.

[CR68] Rendle-Short J (2003). Managing interaction: A conversation analytic approach to the management of interaction by an 8-year-old girl with Asperger’s Syndrome. Issues in Applied Linguistics.

[CR69] Rendle-Short J, Arciuli J, Brock J (2014). Using conversational structure as an interactional resource Children with Asperger’s syndrome and their conversational partners. Communication in Autism.

[CR70] Reznitskaya A, Kuo L-J, Clark A-M, Miller B, Jadallah M, Anderson RC, Nguyen-Jahiel K (2009). Collaborative reasoning: A dialogic approach to group discussions. Cambridge Journal of Education.

[CR71] Rogoff B (1990). Apprenticeship in thinking: Cognitive development in a social context.

[CR72] Sally D, Hill E (2006). The development of interpersonal strategy: autism, theory of mind, cooperation, and fairness. Journal of Economic Psychology.

[CR73] Schegloff EA (2007). Sequence organization in interaction: A primer in conversation analysis.

[CR74] Sidnell J, Stivers T (2013). The Handbook of Conversation Analysis.

[CR75] Sikveland RO, Solem MS, Skovholt K (2021). How teachers use prosody to guide students towards an adequate answer. Linguistics and Education.

[CR76] Solem MS (2016). Negotiating knowledge claims: Students’ assertions in classroom interactions. Discourse Studies.

[CR77] Soter AO, Wilkinson IA, Murphy PK, Rudge L, Reninger K, Edwards MN (2008). What the discourse tells us: Talk and indicators of high-level comprehension. International Journal of Educational Research.

[CR78] Stiegler L (2007). Discovering communication competencies in a nonspeaking child with autism. Language, Speech, and Hearing Services in School.

[CR79] Stivers T, Sidnell J, Stivers T (2012). Sequence organization. The handbook of conversation analysis.

[CR80] Sterponi L, Shankey J (2014). Rethinking echolalia: Repetition as interactional resource in the communication of a child with autism. Journal of Child Language.

[CR81] Sterponi L, Kirky K, Shankey J (2015). Rethinking language in autism. Autism.

[CR82] Sterponi, L. (2017). Language Socialisation and Autism. Language Socialisation, 397–410.

[CR83] Sterponi L, Chen RSY, Pritzker SE, Fenigsen J, Wilce JM (2019). Autism and Emotion: Situation Autistic emotionality in interactional, sociocultural, and political context. The handbook of language and emotion.

[CR84] Stribling P, Rae J, Hyams P, Hae J, Dickerson P (2007). Two forms of spoken repetition in a girl with autism. International Journal of Language and Communication Disorder.

[CR85] Van der Veen C, Van Kruistum C, Michaels S (2015). Productive classroom dialogue as an activity of shared thinking and communicating: A commentary on Marsal. Mind, Culture, and Activity.

[CR86] Willemsen A, Gosen MN, Van Braak M, Koole T, Glopper K (2018). Teachers’ open invitations in whole-class discussions. Linguistics and Education.

[CR87] World Health Organization (1992). The ICD-10 Classification of Mental and Behavioural Disorders: Clinical Descriptions and Diagnostic Guidelines.

[CR88] Zeidan J, Fombonne R, Scorah J, Ibrahim A, Durkin MS, Saxena S, Yusuf A, Shih A, Elsabbagh M (2022). The global prevalence of autism: A systematic review update. Autism Research.

