# Supplementary figures and images for: The Interactional Structure of Accounts During Small Group Discussions Among Autistic Children Receiving Special Education Support in Finland
Source: J Autism Dev Disord. 2023 Feb 7;54(5):1928–46. doi: 10.1007/s10803-023-05916-9 (PMC11136803; doi:10.1007/s10803-023-05916-9)

**Appendix A**

**CA conventions used in this study, according to Jefferson (1986)**

**
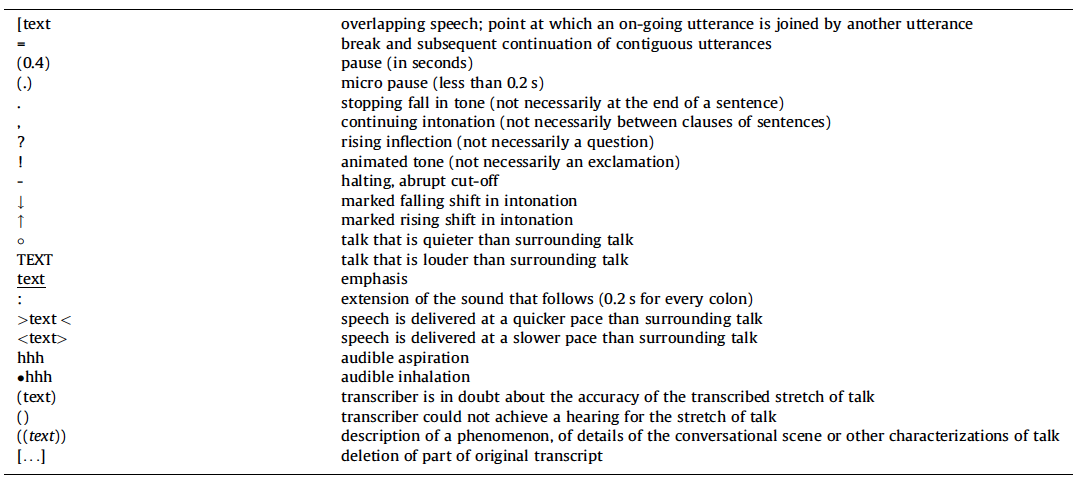
**

Supplement: Supplementary file 1 — Electronic supplementary material 1 (DOCX 113 kb) [file 10803_2023_5916_MOESM1_ESM.docx]
